# Individual‐, household‐, and community‐level factors associated with pregnant married women's discriminatory attitude towards people living with HIV in sub‐Saharan Africa: A multicountry cross‐sectional study

**DOI:** 10.1002/hsr2.430

**Published:** 2021-10-27

**Authors:** Betregiorgis Zegeye, Nicholas Kofi Adjei, Bright Opoku Ahinkorah, Edward Kwabena Ameyaw, Eugene Budu, Abdul‐Aziz Seidu, Sanni Yaya

**Affiliations:** ^1^ HaSET Maternal and Child Health Research Program, Shewarobit Field Office Shewarobit Ethiopia; ^2^ Department of Public Health and Policy University of Liverpool Liverpool UK; ^3^ School of Public Health, Faculty of Health University of Technology Sydney Ultimo New South Wales Australia; ^4^ Department of Population and Health University of Cape Coast Cape Coast Ghana; ^5^ College of Public Health, Medical and Veterinary Sciences James Cook University Townsville Queensland Australia; ^6^ School of International Development and Global Studies University of Ottawa Ottawa Ontario Canada

**Keywords:** DHS, discrimination, factors, global health, HIV/AIDS, sub‐Saharan Africa

## Abstract

**Background and Aims:**

Discriminatory attitude towards people living with human immunodeficiency virus (HIV) remains a major problem in the prevention and treatment of HIV in sub‐Sahara Africa (SSA). Understanding the multiple factors linked to discriminatory attitude towards people living with HIV/AIDS (PLWHA) in SSA is necessary for developing appropriate interventions. This study aimed at investigating the individual, household, and community‐level factors associated with pregnant married women's discriminatory attitude towards people living with HIV/AIDS.

**Methods:**

We used data from the Demographic and Health Surveys of 12 sub‐Saharan African countries conducted between 2015 and 2019. Data on 17 065 pregnant married women were analyzed. Bivariate (chi‐squared test) and multivariable multilevel logistic regression analyses were applied to investigate the factors associated with discriminatory attitude towards PLWHA. The results were reported as adjusted odds ratio (aOR) at 95% confidence interval (CI).

**Results:**

The mean age of participants was 31.2 ± 8.5. The prevalence of discriminatory attitude towards PLWHA was 36.2% (95% CI: 33.4%‐39.1%). Individual/household‐level factors associated with discriminatory attitude towards PLWHA were women's educational level (secondary school‐aOR = 0.49, 95% CI: 0.26‐0.93), husband's educational level (higher education‐aOR = 0.35, 95% CI: 0.16‐0.76), decision‐making power (yes‐aOR = 0.51, 95% CI: 0.38‐0.69), wife‐beating attitude (disagreement with wife beating‐aOR = 0.58, 95% CI: 0.43‐0.79), and religion (Muslim‐aOR = 1.92, 95% CI: 1.22‐3.04). Community socioeconomic status (medium‐aOR = 0.61, 95% CI: 0.41‐0.93) was the only community‐level factor associated with discriminatory attitude towards PLWHA.

**Conclusion:**

More than one‐third of pregnant married women in SSA had discriminatory attitude towards PLWHA. Women's educational level, husband's educational level, decision‐making power, wife‐beating attitude, religion, and community socio‐economic status were associated with discriminatory attitude towards PLWHA. To lessen the prevalence of discriminatory attitude towards PLWHA, considering these significant factors is needed. Therefore, governments and other stakeholders in the respective countries need to increase education coverage. Moreover, empowering women through education and economy is crucial. Finally, working with religious leaders to increase awareness about HIV and discriminatory attitude towards PLWHA should also be a priority in SSA.

**Abbreviations**
CI: confidence interval
DHS: demographic health survey
aOR: adjusted odd ratios
PLWHA: people living with HIV/AIDS
SDGs: sustainable development goals
SSA: sub‐Saharan Africa

## 1 INTRODUCTION

Acquired immune deficiency syndrome (AIDS), which is caused by human immunodeficiency virus (HIV), is a serious public health issue globally,^1^, ^2^, ^3^, ^4^, ^5^ particularly in sub‐Sahara Africa (SSA).^1^, ^2^, ^3^ Nearly 76 million people have been infected, and millions of people have died worldwide since the beginning of the epidemic.^1^ It is estimated that about 37.9 million people are living with HIV/AIDS (PLWHA), and still about 1.7 million new infections were reported globally as of 2019.^1^, ^2^, ^3^, ^4^ HIV is the leading cause of death worldwide (690 000 people died in 2019)^1^, ^5^ and the leading cause of death globally among women of reproductive age (17.3 per 100 000 individuals in 2017).^6^

Globally, approximately 5000 new HIV infections per day are reported,^4^ and about 61% of these new infections occur in SSA.^4^

Although significant progress has been made in recent decades to prevent and control HIV/AIDS,^1^ many people, including women and children, still do not have access to treatment and care.^2^, ^3^ There is some evidence that HIV/AIDS‐related discrimination threatens the effectiveness of prevention and care programmes^7^ which may have a negative impact on victims^8^ who may already be marginalized or stigmatized.^9^ HIV‐related discrimination not only may be directly or indirectly related to a person's perceived or actual HIV status,^10^ but also includes acts or omissions aimed at other key populations and groups at intensified risk of HIV.^10^

Discriminatory attitude is usually attributed to conformist cultural beliefs and practices,^11^ which reflect inadequate knowledge and negative attitude towards PLWHA.^12^ Features of stigma and discrimination towards PLWHA may vary from labeling and discriminatory behavior to negative treatment by family members, friends, healthcare professionals, and communities,^13^, ^14^ which may negatively affect their quality of life.^15^, ^16^, ^17^ Prior studies have shown that fear of stigma and discrimination from families, communities, and health workers are some of the barriers towards the acceptance of HIV testing by pregnant women during antenatal care^18^, ^19^ and prevention of mother to child transmission (PMTCT) care.^20^, ^21^

Although there exist anti‐retroviral treatments for PLWHA,^22^, ^23^ PLWHA are still faced with discrimination and isolation from their families, colleagues, and communities,^22^, ^23^ resulting in job losses and inadequate access and utilization of healthcare services.^23^, ^24^ Since HIV/AIDS‐related stigma and discrimination occur at different levels, including among individuals, between family members, communities, and organizations,^10^, ^16^, ^25^, ^26^ systematic investigations of risk factors at multiple levels is key to designing appropriate policy interventions.^10^, ^16^, ^25^, ^26^

Previous studies in SSA showed that the magnitude of discriminatory attitude towards PLWHA varied from 50% in Nigeria^23^ to about 64.5% in Ethiopia.^27^ There are few studies in Botswana,^28^ Zimbabwe,^29^ Ethiopia,^27^ Nigeria,^23^ and three East African countries.^30^ However, these studies do not reflect recent determinants,^28^ and some are not nationally representative.^29^ This study, therefore, aimed at examining the prevalence of pregnant married women's discriminatory attitude towards PLWHA and its associated factors in SSA using large nationally representative samples.

## 2 METHODS

### 2.1 Data source

We used data from the Demographic and Health Surveys (DHSs) of 12 sub‐Saharan African countries conducted between 2015 and 2019. The DHS is a nationally representative survey that collects data from women of reproductive age (15‐49 years) on several demographic and health indicators, including discriminatory attitude towards PLWHA.^31^ Financial and technical supports for the surveys are usually from the United States Agency for International Development (USAID) and Inner‐City Fund (ICF) international.^32^

The DHSs in the selected countries usually adopt a two‐stage stratified sampling procedure.^33^ In the first stage, Enumeration Areas (EAs) are selected using Probability Proportional to Size (PPS). In the second stage, fixed numbers of households are selected from selected EAs using a systematic sampling technique.^33^ In this study, the countries were selected if the survey was conducted between 2015 and 2019 and included information on all the variables of interest in the study. A total of 17 065 currently pregnant married women from 12 countries were included in the final analysis. The individual recode (IR) files were used, and the datasets are available freely at https://dhsprogram.com/data/available-datasets.cfm. We also followed the guidelines for Strengthening of Observational studies in Epidemiology (STROBE).^34^ Table 1 provides detailed information about selected countries, year of survey, and samples.

**TABLE 1:** List of studied countries, year of survey, and weighted sample (N = 17 065)

SN/Country	Year	Weighted sample	Weighted percentage
1. Angola	2015/16	791	4.63
2. Benin	2017/18	688	4.03
3. Burundi	2016/17	1228	7.20
4. Cameroon	2018/19	899	5.27
5. Ethiopia	2016	973	5.70
6. Guinea	2018	742	4.35
7. Mali	2018	908	5.32
8. Malawi	2015/16	1576	9.24
9. Sierra Leone	2019	786	4.61
10. Zambia	2018/19	855	5.01
11. Zimbabwe	2015	5970	34.98
12. Uganda	2016	1649	9.66
Total		17 065	100.00

### 2.2 Study variables

#### 2.2.1 Outcome variable

The outcome variable was discriminatory attitude towards PLWHA. Two questions were asked to assess discriminatory attitudes towards PLWHA among women who have heard of HIV or AIDS. In the survey, the questions asked were “Should children living with HIV be able to attend school with children who do not have HIV?” and “Would you buy fresh vegetables from a shopkeeper or vendor if you knew that this person had HIV?” Response in the affirmative to either question was considered discriminatory attitudes^35^ and coded “1,” and those who responded “No” were categorized as otherwise and coded “0.”

#### 2.2.2 Explanatory variables

Based on evidence from prior studies, individual/household and community‐level factors were considered for this current study.^10^, ^11^, ^12^, ^13^, ^14^, ^15^, ^16^, ^17^, ^18^, ^19^, ^23^, ^24^, ^25^, ^26^, ^27^, ^28^, ^29^, ^30^

#### 2.2.3 Individual‐/household‐level factors

The individual/household level factors were women's age in years,^15^, ^16^, ^17^, ^18^, ^19^, ^20^, ^21^, ^22^, ^23^, ^24^, ^25^, ^26^, ^27^, ^28^, ^29^, ^30^, ^31^, ^32^, ^33^, ^34^, ^35^, ^36^, ^37^, ^38^, ^39^, ^40^, ^41^, ^42^, ^43^, ^44^, ^45^, ^46^, ^47^, ^48^, ^49^ women's educational status (no formal education, primary school, secondary school, higher), husband's educational status (no formal education, primary school, secondary school, higher), women's occupation (not working, professional/technical/managerial, agricultural, manual, others), economic status (poorest, poorer, middle, richer, richest), media exposure (no, yes), family size (<5, 5+), sex of household head (male, female), religion (Christian, Muslim, Others), antenatal care (ANC) follow‐up (no, yes) decision‐making capacity, and wife‐beating attitude. Regarding decision‐making, we used at least one of the three decision‐making parameters: own health care, large household purchases, and visits to family or relatives, either alone or together with her husband. Responses were coded as “1” if decisions were either made alone or together with the husband on all three of the aforementioned decision‐making parameters, otherwise coded as “0.” Wife‐beating attitude was coded as “0”*—agreed with wife beating* if women justified or accepted wife‐beating norm on at least one of the five wife‐beating parameters (ie, burning food, arguing with him, going out without telling him, neglecting the children and refusing to have sexual intercourse with him). Those who disagreed/not justified with all five parameters were coded as “1”*—disagreed with wife beating*.

#### 2.2.4 Community‐level factors

Place of residence was coded as urban vs rural. Community literacy level (low, medium, high) and community socioeconomic status (low, moderate, high) were calculated as below. The socioeconomic status was computed from occupation, wealth, and education of respondents. We applied principal component analysis to calculate women who were unemployed, uneducated, and poor. A standardized score was derived with a mean score (0) and SD.^1^ The scores were then segregated into tertile 1 (least disadvantaged), tertile 2, and tertile 3 (most disadvantaged) where the least score (tertile 1) denoted greater socioeconomic status with the highest score (tertile 3) denoting lower socioeconomic status. Community literacy level was derived from women who could read and write or not read and write at all.

### 2.3 Statistical analyses

The analysis was carried out as follows. First, descriptive analysis including frequencies and percentages of discriminatory attitude towards PLWHA was calculated and presented using frequency tables and bar charts for the pooled data, and for each country. Thereafter, the Pearson chi‐squared test was conducted to select the candidate explanatory variables using a *P*‐value less than .05 as a cut‐off point. Next, a multicollinearity test was conducted using the variance inflation factor (VIF) for all explanatory variables that had significant associations with the outcome variable. We found no collinearity among the explanatory variables (mean VIF = 1.42, Max = 2.30, Min = 1.02). In the final step, four models were fitted using multilevel binary logistic regression to examine the associations between individual‐/household‐level and community‐level factors and discriminatory attitude towards PLWHA. In all four models, we used the “melogit” Stata command to perform the analysis. Adjusted odds ratios (aOR) at 95% confidence intervals (CI) were estimated. The first model (Model 0), the null model, was fitted to show the variance in discriminatory attitude towards PLWHA, accredited to the clustering of primary sampling units (PSUs). This model had no explanatory and outcome variables. Then, individual‐/household‐level factors (Model I) were included in the second model. In the third model (Model II), community‐level factors were fitted. The last model (Model III) was the complete model that included both the individual‐/household‐ and community‐level factors simultaneously.

The multilevel logistic regression model included both fixed and random effects.^36^, ^37^ The fixed effects (measures of association) show the association between the explanatory variables and discriminatory attitude towards PLWHA, whereas the random effects (measures of variations) were assessed using intra‐cluster correlation (ICC).^38^ The likelihood ratio (LR) test was used to check model adequacy, whereas Akaike's information criterion (AIC) was used to measure how best the different models fitted the data. The “svyset” command was used to account for the complex survey design, including weight, cluster, and strata. The analyses were performed using Stata version‐14 software (Stata Corp, College Station, Texas, USA).

### 2.4 Ethical consideration

For this study, we used publicly available data from DHS. DHS Program is reliable with the standards for ensuring the protection of respondents' privacy. ICF International ensures that the survey complies with the U.S. Department of Health and Human Services regulations for the respect of the right of human subjects, and the respective country institutional review board (IRB) ensured the survey complies with the nation's norms. No further approval was required for this study since the data are secondary and available in the public domain. All data were anonymized prior to the authors receiving the data. More details about data and ethical standards are available at: http://goo.gl/ny8T6X

## 3 RESULTS

### 3.1 Population characteristics

Table 2 shows the background characteristics of the study participants and magnitude of discriminatory attitude across the explanatory variables. A total of 17 065 married pregnant women were included in the analysis. The mean age of participants was 31.2 ± 8.5. Of them, 1805 (15.5%) were in the 15 to 19 years age group, and 9554 (77.1%) were rural residents. Moreover, only 5042 (8.3%) and 4261 (18.3%) of the participants had no formal education and unemployed, respectively. Majority (6445 [89.5%]) of them had no media exposure (ie, newspaper, radio, or television), and 11 445 [82.8%]) were living in male‐headed households. More than half (7495 [56.1%]) of the married pregnant women had no decision‐making power on at least one of the three decision‐making parameters: own health, ability to purchase large household goods, or visits to family or relative. In addition, 6507 (51.8%) of the respondents accepted or justified wife beating for at least one of the five reasons: burning food, neglecting the children, arguing with husband, going out without telling husband, and refusing to have sexual intercourse with husband.

**TABLE 2:** Prevalence of discriminatory attitude towards PLWHA among married pregnant women (N = 17 065): Evidence from 12 sub‐Saharan African countries DHSs

Variables	Number (Weighted %)	Discriminatory attitude		Chi‐square, *P*‐value
		No, Freq./perc.	Yes, Freq./perc.	
Overall prevalence	17 065 (36.2)			
Age in years				χ2 = 25.02, *P* < .001
15‐19	1805 (15.4)	762 (50.00)	762 (50.00)	
20‐24	3608 (32.9)	1773 (56.41)	1370 (43.59)	
25‐29	3441 (20.6)	1626 (54.47)	1359 (45.53)	
30‐34	2528 (18.9)	1260 (56.50)	970 (43.50)	
35‐39	1451 (8.6)	674 (53.07)	596 (46.93)	
40‐44	476 (2.6)	216 (54.27)	182 (45.73)	
45‐49	100 (1.0)	36 (44.44)	45 (55.56)	
Women's educational status				χ2 = 1.10, *P* < .001
No formal education	5042 (8.3)	1301 (34.13)	2511 (65.87)	
Primary school	4952 (62.0)	2720 (59.27)	1869 (40.73)	
Secondary school	2971 (23.5)	1950 (69.77)	845 (30.23)	
Higher	444 (6.2)	376 (86.44)	59 (13.56)	
Husband's educational status				χ2 = 794.76, *P* < .001
No formal education	4544 (9.2)	1228 (35.35)	2246 (64.65)	
Primary school	4257 (52.4)	2305 (59.50)	1569 (40.50)	
Secondary school	3721 (28.3)	2205 (63.84)	1249 (36.16)	
Higher	887 (10.1)	609 (73.46)	220 (26.54)	
Women's occupation				χ2 = 135.69, *P* < .001
Not working	4261 (18.3)	1920 (52.53)	1735 (47.47)	
Professional/technical/managerial	467 (7.3)	348 (80.37)	85 (19.63)	
Agricultural	4852 (43.6)	2286 (53.01)	2026 (46.99)	
Manual	998 (14.3)	512 (59.60)	347 (40.40)	
Others	2830 (16.5)	1280 (53.99)	1091 (46.01)	
Economic status				χ2 = 428.63, *P* < .001
Poorest	3143 (22.3)	1145 (43.87)	1465 (56.13)	
Poorer	2927 (20.9)	1215 (48.72)	1279 (51.28)	
Middle	2590 (20.2)	1186 (52.95)	1054 (47.05)	
Richer	2456 (17.5)	1282 (59.02)	890 (40.98)	
Richest	2293 (19.1)	1519 (71.82)	596 (28.18)	
Media exposure				χ2 = 35.72, *P* < .001
No	6445 (89.5)	2846 (51.66)	2663 (48.34)	
Yes	6964 (10.5)	3501 (57.19)	2621 (42.81)	
Family size				χ2 = 76.74, *P* < .001
<5	5826 (49.6)	3044 (59.11)	2106 (40.89)	
5+	7583 (50.4)	3303 (50.96)	3178 (49.04)	
Sex of household head				χ2 = 9.73, *P* < .01
Male	11 445 (82.8)	5345 (53.97)	4559 (46.03)	
Female	1964 (17.2)	1002 (58.02)	725 (41.98)	
Decision‐making				χ2 = 280.28, *P* < .001
No	7495 (56.1)	3001 (47.48)	3319 (52.52)	
Yes	5914 (43.9)	3346 (63.00)	1965 (37.00)	
Wife‐beating attitude				χ2 = 353.47, *P* < .001
Agreed with wife beating	6507 (51.8)	2639 (45.81)	3122 (54.19)	
Disagreed with wife beating	6902 (48.2)	3708 (63.17)	2162 (36.83)	
Religion				χ2 = 1.10, *P* < .001
Christian	8354 (86.1)	5008 (65.78)	2605 (34.22)	
Muslim	4525 (12.7)	1207 (32.79)	2474 (67.21)	
Others	530 (1.2)	132 (39.17)	205 (60.83)	
ANC F/UP				χ2 = 177.02, *P* < .001
No	1150 (1.7)	244 (30.69)	551 (69.31)	
Yes	8149 (98.3)	4008 (55.50)	3213 (44.50)	
Place of residence				χ2 = 109.91, *P* < .001
Urban	3855 (22.9)	2087 (62.17)	1270 (37.83)	
Rural	9554 (77.1)	4260 (51.49)	4014 (48.51)	
Community literacy level				χ2 = 386.37, *P* < .001
Low	4232 (32.0)	2159 (45.95)	2540 (54.05)	
Medium	3829 (33.8)	2083 (53.89)	1782 (46.11)	
High	3318 (34.2)	2105 (68.63)	962 (31.37)	
Community socioeconomic status				χ2 = 489.55, *P* < .001
Low	5471 (43.2)	2736 (45.19)	3318 (54.81)	
Medium	2574 (21.4)	1433 (59.78)	964 (40.22)	
High	3334 (35.4)	2178 (68.49)	1002 (31.51)	

Abbreviations: ANC F/UP, antenatal care follow‐up; NA, not applicable.

### 3.2 Distribution of discriminatory attitude across explanatory/control variables

The prevalence of discriminatory attitude towards PLWHA by explanatory variables and subgroups is shown in Table 2. The prevalence varied across the explanatory variables. For instance, discriminatory attitude was found to be higher among pregnant married women with no formal education (65.9%) than those with higher education (13.6%). We further observed a higher prevalence (52.5%) among those with no decision‐making power than those with decision‐making power (37.0%). A higher prevalence of discriminatory attitude was also found among those who accepted or justified wife beating (54.2%).

### 3.3 Prevalence of discriminatory attitude

**FIGURE 1:**
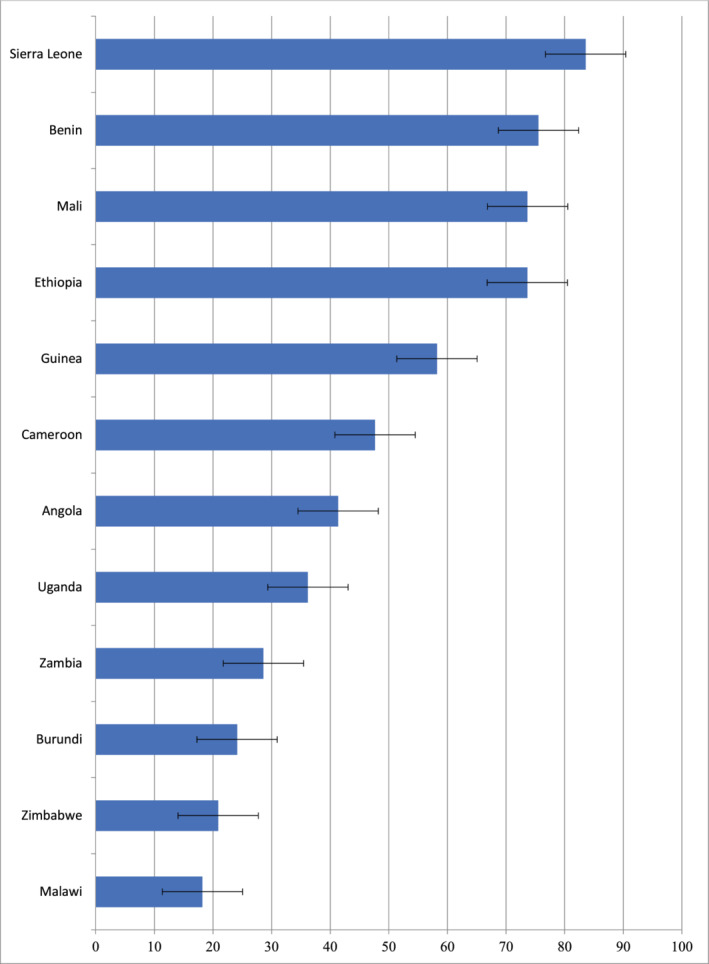
Prevalence of discriminatory attitude towards PLWHA among married pregnant women across studied countries: Evidence from 12 SSA countries DHSs

Overall, more than one‐third (36.2%, 95% CI: 33.4%‐39.1%) of respondents in the selected countries had discriminatory attitude towards PLWHA. We however found cross‐country differences in the prevalence of discriminatory attitude towards PLWHA. Figure 1 shows the prevalence of discriminatory attitude towards PLWHA among married pregnant women across the studied countries. Sierra Leone reported the highest prevalence (83.6%) followed by Benin (75.6%), and Ethiopia (73.6%). The lowest prevalence was reported in Malawi (18.2%), Zimbabwe (20.9%), and Burundi (24.1%).

### 3.4 Fixed effect results (measures of association)

Table 3 shows the fixed effects results of the individual‐/household‐ and community‐level factors associated with married pregnant women's discriminatory attitude towards PLWHA. The results showed that women who had secondary education were less likely to report discriminatory attitude (aOR = 0.49, 95% CI: 0.26‐0.93) compared to those with no formal education. Similarly, husband's educational level was strongly associated with discriminatory attitude, where those who completed primary education (aOR = 0.38, 95% CI: 0.23‐0.63), secondary education (aOR = 0.29, 95% CI: 0.16‐0.51), and higher education (aOR = 0.35, 95% CI: 0.16‐0.76) had lower odds of reporting discriminatory attitude than those with no formal education. Religion was found to be associated with discriminatory attitude towards PLWHA. Muslim married pregnant women had higher odds (aOR = 1.92, 95% CI: 1.22‐3.04) of reporting discriminatory attitude than Christians.

**TABLE 3:** Multilevel multivariable logistic regression results of the individual‐/household‐ and community‐level factors associated with discriminatory attitude towards PLWHA among married pregnant women (N = 17 065): Evidence from 12 sub‐Saharan African countries DHSs

		Model I	Model II	Model III
Variables	Model 0	aOR [95% CI)	aOR [95% CI)	aOR [95% CI)
Age in years				
15‐19		1.29 (0.72‐2.33)		
20‐24		0.97 (0.52‐1.82)		1.31 (0.73‐2.35)
25‐29		1.14 (0.60‐2.17)		0.98 (0.52‐1.83)
30‐34		0.96 (0.46‐1.99)		1.16 (0.61‐2.20)
35‐39		0.90 (0.30‐2.63)		0.97 (0.47‐2.01)
40‐44		2.84 (0.45‐17.59)		0.97 (0.33‐2.84)
45‐49		1.29 (0.72‐2.33)		2.98 (0.49‐18.06)
Women's educational status				
No formal education				
Primary school		0.72 (0.44‐1.18)		0.77 (0.47‐1.27)
Secondary school		0.44 (0.24‐0.83)*		0.49 (0.26‐0.93)*
Higher		0.48 (0.15‐1.53)		0.56 (0.17‐1.78)
Husband's educational status				
No formal education				
Primary school		0.37 (0.22‐0.62)***		0.38 (0.23‐0.63)***
Secondary school		0.29 (0.16‐0.52)***		0.29 (0.16‐0.51)***
Higher		0.36 (0.17‐0.76)**		0.35 (0.16‐0.76)**
Women's occupation				
Not working				
Professional/technical/managerial		0.70 (0.29‐1.69)		0.75 (0.31‐1.83)
Agricultural		1.36 (0.89‐2.06)		1.38 (0.91‐2.10)
Manual		1.01 (0.61‐1.67)		1.02 (0.62‐1.68)
Others		0.82 (0.47‐1.43)		0.89 (0.51‐1.57)
Economic status				
Poorest				
Poorer		0.89 (0.60‐1.32)		0.98 (0.66‐1.46)
Middle		0.76 (0.50‐1.18)		0.91 (0.59‐1.42)
Richer		0.62 (0.37‐1.03)		0.81 (0.48‐1.37)
Richest		0.60 (0.31‐1.15)		1.03 (0.47‐2.25)
Media exposure				
No				
Yes		0.66 (0.31‐1.41)		0.68 (0.32‐1.44)
Family size				
<5				
5+		1.13 (0.82‐1.57)		1.08 (0.78‐1.49)
Sex of household head				
Male				
Female		1.31 (0.88‐1.95)		1.32 (0.89‐1.95)
Religion				
Christian				
Muslim		1.98 (1.25‐3.14)**		1.92 (1.22‐3.04)**
Others		0.72 (0.14‐3.61)		0.66 (0.13‐3.29)
Wife‐beating attitude				
Agreed with wife beating				
Disagreed with wife beating		0.55 (0.41‐0.75)***		0.58 (0.43‐0.79)***
Decision‐making				
No				
Yes		0.52 (0.38‐0.70)***		0.51 (0.38‐0.69)***
Antenatal care follow‐up				
No				
Yes		1.63 (0.56‐4.72)		1.70 (0.59‐4.87)
Place of residence				
Urban				
Rural			1.07 (0.71‐1.62)	1.12 (0.65‐1.92)
Community educational level				
Low				
Medium			0.59 (0.43‐0.81)**	0.78 (0.53‐1.12)
High			0.41 (0.27‐0.63)***	0.62 (0.36‐1.04)
Community socioeconomic status				
Low				
Medium			0.65 (0.46‐0.91)*	0.61 (0.41‐0.93)*
High			0.52 (0.34‐0.80)**	0.70 (0.39‐1.25)

Abbreviations: aOR, adjusted odds ratios; Ref, reference.

*** *P* < .001;

** *P* < .01;

* *P* < .05.

Furthermore, we found lower odds of discriminatory attitude towards PLWHA among those who had decision‐making power (aOR = 0.51, 95% CI: 0.38‐0.69) compared to those with no decision‐making power. Married pregnant women who did not accept wife beating were less likely to report discriminatory attitude (aOR = 0.58, 95% CI: 0.43‐0.79) compared to those who justified or accept wife beating.

Regarding community‐level factors, we observed lower odds of discriminatory attitude among married pregnant women living in communities of medium socioeconomic status (aOR = 0.61, 95% CI: 0.41‐0.93) as compared to those living in communities of low socioeconomic status (Table 3).

### 3.5 Random effect results (measures of variation)

The random effects models of the individual‐/household‐ and community‐level factors associated with married pregnant women's discriminatory attitude towards PLWHA are shown in Table 4. The AIC estimate was lower in the complete model (Model III) indicating a best‐fitted model. The ICC in the empty model (ICC = 0.22) showed that the odds of discriminatory attitude towards PLWHA among currently pregnant married women varied across clusters (σ2 = 0.97, 0.61‐1.54). The ICC estimate model in the empty model (22%) decreased by 6% in model I (ICC = 16%), 1% in model II (ICC = 15%) and again by 1% in model III (ICC = 14%), which had both individual‐/household‐ and community‐level factors. These estimates showed that the variations in the likelihood of reporting discriminatory attitude towards PLWHA can be attributed to the variances in the clustering at the primary sampling units (Table 4).

**TABLE 4:** Random effect results (measure of variation) results of the individual‐/household‐ and community‐level factors associated with discriminatory attitude towards PLWHA among married pregnant women (N = 17 065): Evidence from 12 sub‐Saharan African countries DHSs

Random effect result	Model 0	Model I	Model II	Model III
PSU variance (95% CI)	0.97 (0.61‐1.54)	0.63 (0.28‐1.42)	0.60 (0.33‐1.10)	0.56 (0.23‐1.36)
ICC	0.22	0.16	0.15	0.14
LR Test	45.42	11.02	21.47	8.70
Wald chi‐square and *P*‐value	Ref	106.66	67.55	113.33
Model fitness				
Log‐likelihood	−1057.26	−688.73	−1021.86	−681.95
AIC	2118.52	1437.466	2057.72	1433.91
N	17 065	17 065	17 065	17 065

Abbreviations: AIC, Akaike information criterion; ICC, intra‐class correlation coefficient; LR, likelihood ratio; N, total observation.

## 4 DISCUSSION

We investigated the prevalence of discriminatory attitude towards PLWHA among pregnant married women in 12 countries in SSA and examined associated individual‐, household‐, and community‐level factors. The study shows that 36.2% (95% CI: 33.4%‐39.1%) of pregnant married women had discriminatory attitude towards PLWHA. Both individual‐/household‐ and community‐level factors were found to be linked with discriminatory attitude.

Regarding the individual/household factors, we found that pregnant married women who had higher educational levels were less likely to have discriminatory attitude compared to those with no formal education.^11^, ^19^, ^21^ Prior studies have indicated that educated women have better knowledge about HIV transmission and prevention than non‐educated women,^11^, ^39^, ^40^, ^41^ because knowledge and education may increase an individual's protective behavior in eliminating myths and discrimination,^11^ even HIV/AIDS‐related discrimination.^11^ Evidence shows that education can increase knowledge and changes attitude towards HIV/AIDS in different mechanisms.^42^, ^43^ Education facilitates transferring of information about HIV/AIDS through increasing exposure to information; could it be by formal education, mass media, or other channels.^44^, ^45^ In addition, formal education can change the thought process of individuals and enable them to recognize and evaluate their knowledge and behaviors with respect to HIV/AIDS transmission or prevention.^45^ On the other hand, the lack of knowledge about transmission and prevention of HIV/AIDS leads the individual to accept myths and non‐factual information about HIV,^11^, ^27^ which leads to having discriminatory attitude towards PLWHA.^27^ Similarly, lower odds of discriminatory attitude were observed among educated husbands as found in previous studies in Kuwait^46^ and Bangladesh.^47^

The findings revealed a strong association between religion and discriminatory attitude towards PLWHA.^48^ We found that pregnant married Muslim women were more likely to have discriminatory attitudes towards PLWHA than Christians. A prior study in Ethiopia shows comparable finding.^43^ This finding may be attributed to the low prevalence of HIV among Muslims,^49^ due to their strict religious,^49^ which may reduce risky sexual behaviors, homosexuality, and drug use.^49^ A study conducted in Senegal among Muslim and Catholic followers showed that people who consider religion as sacred and important are more likely to show HIV discriminatory attitude compared to individuals who attach less importance to religion.^50^ Therefore, taking into consideration of this and related evidence as well as working with religious leaders to achieve better results related to reduction of the relatively higher prevalence of discriminatory attitude towards PLWHA among these populations is essential.^43^, ^49^, ^50^

As previously observed,^11^, ^43^ decision‐making power was strongly associated with the odds of discriminatory attitude towards PLWHA, where women who had decision‐making power had lower odds of having discriminatory attitude than those with no decision‐making power. This finding can be attributed to the fact that women who have decision‐making power are usually educated^11^ and more knowledgeable about HIV/AIDS‐related issues.^11^ The study conducted in South Asian migrant women shows association between women's decision‐making power and knowledge about HIV.^51^ More specifically, the study found higher odds of having information about HIV/AIDS among women with higher decision‐making power.^51^ Hence, enhancing women's decision‐making power through education and paid employment,^52^ needs to be considered important in reducing the prevalence of discriminatory attitude towards PLWHA. Knowledge about HIV/AIDS has been shown in other studies to be important in reducing myths and perceptions.^53^, ^54^

Consistent with prior studies in Zimbabwe,^29^ Ghana,^55^ and other two African counties^56^ and three East African countries,^30^ we found that community socioeconomic status is strongly associated with discriminatory attitude towards PLWHA. More specifically, the study shows lower odds of discriminatory attitude towards PLWHA among currently pregnant married women living in communities of medium socioeconomic status compared to those living in communities of low socioeconomic status. A plausible reason could be that women living in greater socioeconomic communities had better opportunities of accessing media and educational achievement as compared to women living in households or communities of lower socioeconomic status.^57^, ^58^ Because of the capacities to afford travel costs,^43^ wealthier women are more likely to travel to community gatherings, health centers, hospitals, and urban centers where better information exposure are available^43^ that would enable them to acquire knowledge and promote positive attitude towards PLWHA. Different scholars documented that unable to afford transportation costs is the main factor to not access health information even the services.^59^, ^60^

### 4.1 Strengths and limitations of the study

This study has some strengths and limitations. One of the strengths is that we used nationally representative data to investigate individual‐, household‐, and community‐level factors associated with discriminatory attitude towards PLWHA. Nonetheless, the study has the following limitations. First, the findings may not represent all SSA countries since our analysis was based on only 12 countries. Second, recall bias may affect the findings, due to our reliance on self‐reported data.^33^ Finally, the cross‐sectional nature of the study design may not permit concluding causal‐effect relationship.

## 5 CONCLUSION

More than one‐third of pregnant married women in SSA had discriminatory attitude towards PLWHA. While women's educational level, husband educational level, decision‐making, women's attitude towards domestic violence, and religion were pragmatic as important individual‐/household‐level factors associated with discriminatory attitude towards PLWHA, community socioeconomic status was the only community‐level factors shown to be significantly associated with discriminatory attitude. To lessen the prevalence of discriminatory attitude towards PLWHA, considering these significant factors are needed. Therefore, governments and other stakeholders in the respective countries essential to increase education coverage. Moreover, empowering women through education and economy is crucial. Finally, working with religious leaders to increase awareness about HIV and discriminatory attitude towards PLWHA should also be a priority in SSA.

## FUNDING

No funding was received for this study.

## CONFLICT OF INTEREST

The authors declare there is no conflict of interest.

## AUTHOR CONTRIBUTION

Conceptualization: Sanni Yaya, Betregiorgis Zegeye.

Formal Analysis: Sanni Yaya, Betregiorgis Zegeye.

Investigation: Sanni Yaya.

Writing ‐ Original Draft Preparation: Sanni Yaya, Betregiorgis Zegeye.

Writing ‐ Review & Editing: Nicholas Kofi Adjei, Bright Opoku Ahinkorah, Edward Kwabena Ameyaw, Eugene Budu, Abdul‐Aziz Seidu.

All authors have read and approved the final version of the manuscript.

Sanni Yaya had full access to all of the data in this study and takes complete responsibility for the integrity of the data and the accuracy of the data analysis.

## TRANSPARENCY STATEMENT

Sanni Yaya affirms that the manuscript is an honest, accurate, and transparent account of the study being reported, that no important aspects of the study have been omitted, and that any discrepancies from the study as planned have been explained.

## Data Availability

All analyzed data are freely available to the public through www.measuredhs.com.
